# Melatonin-Mediated Molecular Responses in Plants: Enhancing Stress Tolerance and Mitigating Environmental Challenges in Cereal Crop Production

**DOI:** 10.3390/ijms25084551

**Published:** 2024-04-21

**Authors:** Ihsan Muhammad, Shakeel Ahmad, Weijun Shen

**Affiliations:** 1 Guangxi Key Laboratory of Forest Ecology and Conservation, State Key Laboratory for Conservation and Utilization of Agro-Bioresources, College of Forestry, Guangxi University, Nanning 530004, China; ihsanagrarian@yahoo.com; 2 State Key Laboratory for Conservation and Utilization of Subtropical Agro-Bioresources, College of Life Science and Technology, Guangxi University, Nanning 530004, China; shakeel1287@hotmail.com

**Keywords:** molecular regulation, heavy metal stress, drought stress, antioxidant defense, sustainable agriculture, melatonin

## Abstract

Cereal crops are crucial for global food security; however, they are susceptible to various environmental stresses that significantly hamper their productivity. In response, melatonin has emerged as a promising regulator, offering potential benefits for stress tolerance and crop growth. This review explores the effects of melatonin on maize, sorghum, millet, rice, barley, and wheat, aiming to enhance their resilience to stress. The application of melatonin has shown promising outcomes, improving water use efficiency and reducing transpiration rates in millet under drought stress conditions. Furthermore, it enhances the salinity and heavy metal tolerance of millet by regulating the activity of stress-responsive genes. Similarly, melatonin application in sorghum enhances its resistance to high temperatures, low humidity, and nutrient deficiency, potentially involving the modulation of antioxidant defense and aspects related to photosynthetic genes. Melatonin also exerts protective effects against drought, salinity, heavy metal, extreme temperatures, and waterlogging stresses in maize, wheat, rice, and barley crops by decreasing reactive oxygen species (ROS) production through regulating the antioxidant defense system. The molecular reactions of melatonin upregulated photosynthesis, antioxidant defense mechanisms, the metabolic pathway, and genes and downregulated stress susceptibility genes. In conclusion, melatonin serves as a versatile tool in cereal crops, bolstering stress resistance and promoting sustainable development. Further investigations are warranted to elucidate the underlying molecular mechanisms and refine application techniques to fully harness the potential role of melatonin in cereal crop production systems.

## 1. Introduction

Cereal crops, including maize (*Zea mays*), wheat (*Triticum aestivum*), rice (*Oryza sativa*), barley (*Hordeum vulgare* L.), millet (*Panicum miliaceum* L.), and sorghum (*Sorghum bicolor*), play a vital role in global agriculture due to their significant productivity and adaptability in the food and feed industries [1,2,3,4,5,6]. These crops not only serve as a primary source of income for millions of farmers worldwide [3,7,8] but also have extensive applications in biofuels, industrial materials, and other non-food products, thereby contributing to economic growth and sustainability [9,10,11]. Among these crops, maize is notably one of the most extensively cultivated across diverse agro-ecological zones [3,12,13,14,15]. However, the productivity and sustainability of these crops are challenged by several abiotic stresses, including drought, salinity, high and low temperature, pesticide exposure, and heavy metal contamination, which threaten global food security [4,16,17,18]. Pesticides influence crop yield and development globally by protecting plants from pests and diseases, thereby enhancing agricultural productivity and food security [19,20].

Melatonin, initially discovered for its role in animal circadian rhythm regulation [21], recently gained recognition for involvement in plant growth, development, and physiological processes, such as seed germination, root development, and stress tolerance [22,23,24,25,26]. Unlike its synthesis in animals, plant melatonin synthesis follows a unique pathway beginning with the amino acid tryptophan and leading to serotonin via distinct enzymes. This process, sensitive to environmental cues, underscores the potential role of melatonin in bolstering plant resilience against abiotic stresses [27]. In plants, tryptophan converts into tryptamine by the enzyme tryptophan decarboxylase (TDC) and subsequently into serotonin via tryptamine 5-hydroxylase (T5H) [28]. Following this, serotonin undergoes acetylation and methylation by serotonin N-acetyltransferase (SNAT) and *N*-acetylserotonin methyltransferase (ASMT), respectively. The responsiveness of this pathway to environmental cues underscores its adaptability, positioning melatonin as a pivotal molecule within complex signaling networks that enhance stress resilience by interacting with phytohormonal pathways. Understanding melatonin’s biosynthesis, perception, and signaling mechanisms is crucial for its potential in enhancing stress adaptation and improving crop resilience [27]. Furthermore, melatonin enhances plant stress tolerance by modulating stress-responsive genetic pathways, thereby improving plant resilience and productivity under stress conditions [29,30,31,32,33].

**Figure 1:**
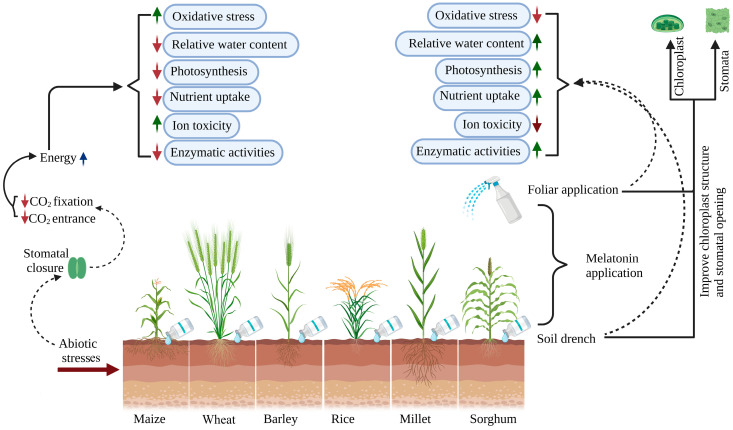
Effect of melatonin application on oxidative stress, relative water content, photosynthesis, nutrient uptake, ion toxicity, enzymatic activities, stomatal traits, chloroplast structure, and enhanced tolerance in cereal crops under abiotic stress. Red arrows indicate downregulation, and green arrows indicate upregulation of physiological and biochemical processes.

This review delves into melatonin’s broad effect on plant physiology, with a focus on its impact on growth, antioxidant enzyme activities, nutrient uptake, photosynthesis capacity, and gene expression regulation (Figure 1). We aim to elucidate the mechanisms through which melatonin mitigates stress, thereby enhancing crop production under adverse environmental conditions. Exposure to abiotic stress often leads to increased oxidative stress and ion toxicity, markedly reducing plant water content, photosynthesis, nutrient uptake, and enzyme activity. In contrast, melatonin application exhibited a significant ameliorative effect, notably elevated relative water content, photosynthesis, nutrient uptake, and enzyme activity while simultaneously reducing oxidative stress (Figure 1).

Recent studies have highlighted the role of exogenous melatonin in improving crop tolerance to a variety of environmental stresses, including drought [29,34], salinity [35,36], and heavy metal toxicity [37,38]. Melatonin application positively influences plant growth and yield by enhancing photosynthesis, boosting antioxidant activity, and regulating gene expression [23,37,39,40]. These enhancements contribute significantly to crop development and enzymatic activities, although the precise mechanisms remain to be fully elucidated.

**Figure 2:**
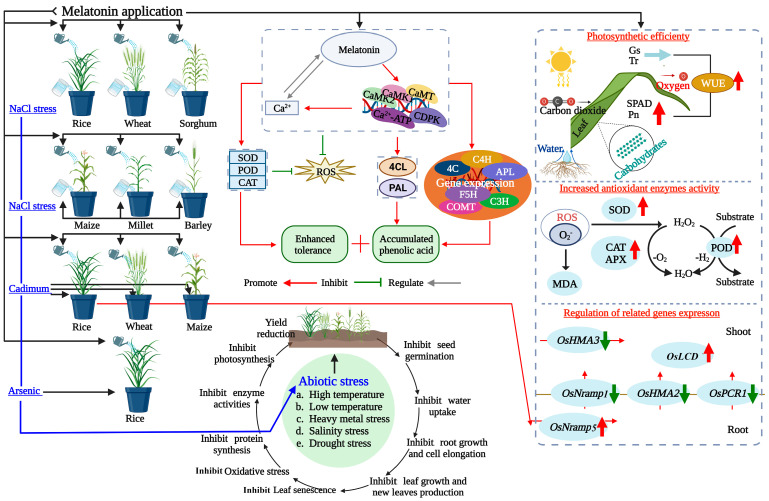
Melatonin and Ca^2+^ affected phenolic acid biosynthesis and enhanced tolerance in barley under NaCl stress. The schematic model shows the effect of melatonin on heavy metal accumulation, toxicity, and salinity stress in cereal crops. Melatonin treatment increased the activity of antioxidative enzymes (SOD, POD, CAT, and APX) and decreased the content of MDA in leaves. Additionally, it regulated the expression of cadmium (Cd) transport genes, namely *OsNramp1*, *OsNramp5*, *OsHMA2*, *OsHMA3*), *OsLCD*, and *OsPCR1*, associated with Cd transport in response to heavy metal stress in both roots and shoots. Green arrows indicate downregulation, and red arrows indicate upregulation of the respective pathways and gene expressions. MDA: malondialdehyde; CAT: catalase; SOD: superoxide dismutase; POD: peroxidase; APX: ascorbate peroxidase; PAL: phenylalanine ammonia-lyase; 4CL: 4-coumarate-CoA ligase; ROS: reactive oxygen species.

Exposure to pesticides, such as organophosphates, organochlorines, and carbamates, has been associated with various metabolic disturbances, including oxidative stress and changes in glucose and lipid metabolism [41]. In addition, pesticides play a dual role in crop production: they are crucial for managing pests and diseases but can also act as abiotic stressors, potentially disrupting plant physiological and biochemical processes [42]. While protecting crops from external biotic stress, residues from pesticide use may adversely affect biochemical pathways and alter the metabolic profile of crops [43]. Meanwhile, the pivotal role of organic biostimulators is in bolstering the metabolic responses and antioxidant defense against pests, pathogens, pesticides, and the resilience of wheat under diverse environmental conditions [43]. This complexity highlights the nuanced impact of pesticide usage in agriculture on crop health and quality while also highlighting the promising potential of melatonin to counteract the detrimental effects of pesticides and enhance the biochemical composition of crops [42,44]. Its roles as a biostimulator and modulating agent in responses to abiotic and biotic stresses are crucial in enhancing plant resilience [44,45]. Despite numerous efforts to mitigate the adverse effects of pesticides [18,42], an effective solution to combat pesticide-related hazards has yet to be discovered. Delving into the role of melatonin in agriculture could significantly advance our understanding of its potential to improve grain quality for human consumption. The role of melatonin in modulating gene expression is complex and varies across cereal species, particularly under stress conditions [46,47]. Its crucial role in regulating gene expression significantly influences cellular growth, stress adaptation, and hormonal signaling in cereal crops, underscoring its importance in plant development and resilience strategies [48,49] (Figure 2).

Melatonin application to maize seedlings has been shown to activate genes associated with photosynthesis and antioxidant defense during drought stress [29,30], effectively decreasing ROS levels. This effect is associated with stronger antioxidant defense, including the upregulation of glutathione-ascorbate (GSH-AsA) related genes in wheat [50], and the modulation of physiology and waxy genes in barley [51]. Melatonin improved the accumulation of proline and osmolytes, crucial for stress tolerance, by regulating the expression of genes such as *Oryza sativa pyrroline-5-carboxylate synthase* (*OsP5CS*), *Oryza sativa sucrose synthase 7* (*OsSUS7*), and *Oryza sativa sucrose phosphate synthase 1* (*OsSPS1*), thereby boosting chlorophyll synthesis [52]. These genetic changes lead to improved physiological and biochemical responses, improving the resilience of maize plants to drought. Furthermore, melatonin coupled with N fertilization significantly improved the gene expression and nutrient uptake in maize under Cd stress [31].

In addition to modulating gene expression, melatonin also influences the key antioxidant enzymes like ascorbate peroxidase (APX), superoxide dismutase (SOD), catalase (CAT), and peroxidase (POD) in cereal crops, countering ROS accumulation induced by environmental stressors like superoxide radicals (O_2_^−^) and hydrogen peroxide (H_2_O_2_), thereby mitigating oxidative damage [15,29,30]. Ahmad et al. [29] demonstrated that melatonin application improved CAT and SOD in maize seedlings under drought, and when coupled with arbuscular mycorrhizal fungi, it reduced the effects of drought stress and increased antioxidant enzyme activities in tobacco seedlings [53]. Moreover, melatonin and arbuscular mycorrhizal inoculation synergistically mitigate heat-induced leaf senescence in cool-season plants by enhancing photosynthesis and growth, modulating phytohormonal levels, and reducing oxidative stress [54].

Moreover, melatonin’s interaction with plant hormonal signaling pathways alters cereal crop growth, affecting hormones such as auxin, abscisic acid (ABA), and gibberellins (GAs). A previous study showed that melatonin biosynthesis in *Arabidopsis thaliana* increases the response to ABA and salinity stress, indicating melatonin’s involvement in ABA-regulated stress reactions [23]. It is also proposed that melatonin might interact with phytohormones like GAs and cytokinins to regulate nutrient uptake and balance in cereal crops under harsh environmental conditions [55].

Melatonin is increasingly recognized for its diverse and significant role in promoting the development and stress tolerance of cereal crops. Its influence on gene expression, antioxidant activities, and hormonal signaling pathways collectively underscores its potential in improving crop growth under stress conditions. However, despite the promising advancement in understanding melatonin’s multifaceted roles, a comprehensive delineation of the precise molecular mechanisms underlying its effect across various stress responses in different crop species remains elusive. This gap in knowledge underscores the need for our study, which aims to dissect the intricate relationship between melatonin, stress-related genes, and metabolic pathways in cereal crops. Additional research is required to clarify the most effective techniques and concentrations for the application of melatonin in order to promote sustainable and environment-friendly farming practices.

## 2. Effects of Melatonin on Stress Tolerance in Cereal Crops

Melatonin significantly increases stress tolerance in maize, millet, and sorghum, both against biotic and abiotic stresses. Its application improves early growth, biochemical, and physiological attributes of plants exposed to cadmium (Cd) stress [31]. Moreover, melatonin increases hydraulic conductance, mitigating drought stress in cereal crops, and significantly improves antioxidant enzyme activities, crop growth, and photosynthetic capacity under such conditions [29,30]. The combined use of melatonin with N fertilization exhibited a synergistic effect in encouraging stress tolerance in maize [31].

In cereal cops, melatonin plays a multifaceted role in enhancing stress tolerance, influencing key physiological pathways including growth regulation and water management and with the activation of antioxidant defense mechanisms (Figure 2). It regulates stress-responsive gene expression, including those involved in ABA signaling, osmotic adjustment, and photosynthesis, thereby enhancing stress tolerance [23]. Furthermore, melatonin improves water uptake and transport efficiency under stressful water conditions by increasing hydraulic conductivity and promoting root development [30]. Its influence extends across various physiological and molecular pathways, bolstering the resilience of cereal crops under stress conditions.

Abiotic stress such as drought, salinity, high and low temperature, heavy metals, and xenobiotic toxicity negatively affects crop growth, development, and productivity [55,56,57,58,59,60]. These stressors disrupt water balance, interfere with photosynthesis, and trigger ROS accumulation, damaging cellular structures [49,61,62]. The previous literature showed that exogenous melatonin enhances plant growth and physiological attributes under Cd and drought stress in maize [30,31,63]. Recent research highlights priming-mediated abiotic stress management, where pre-exposure to mild stress improves plant tolerance to subsequent stress events [64,65]. Studies by Yuan et al. [4] and Bhowal et al. [23] have explored transcriptome and physiological responses to drought in different millet genotypes and the role of serotonin and melatonin biosynthesis in stress and development, respectively. Innovations such as exogenous melatonin, nanoparticles, and priming-mediated strategies show promise in improving crop growth and stress tolerance, potentially increasing crop productivity and sustainability [40,64].

### 2.1. Maize

**Figure 3:**
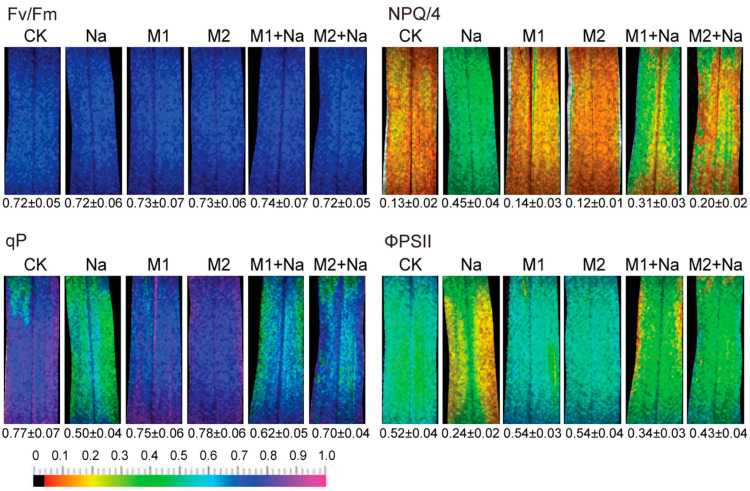
The effects of melatonin on the maximum efficiency of PSII photochemistry (Fv/Fm); non-photochemical quenching coefficient (NPQ), photochemical quenching and ΦPSII (qP). and quantum yield of PSII electron transport (ΦPSII) in maize seedlings under salinity stress. Quantitative mean values ± SD (n = 3) are shown below the individual fluorescence images. The legend is as follows: control, 0 NaCl + 0 melatonin (CK), 150 mM NaCl + 0 melatonin (Na), 0 NaCl + 20 μM melatonin (M1), 0 NaCl + 100 μM melatonin (M2), 150 mM NaCl + 20 μM melatonin (M1 + Na), and 150 mM NaCl + 100 μM melatonin (M2 + Na). Reproduced with the permission of [68] © (2018) Wiley.

Melatonin positively impacts maize growth under various stress conditions, including drought, Cd, salinity, temperature, etc. Ma et al. [31] investigated the effects of exogenous melatonin and N application on maize under Cd stress and revealed notable enhancements in root length, volume, and biomass, alongside a reduction in Cd accumulation. Furthermore, melatonin not only improves hydraulic conductance and reduces oxidative damage in maize during a drought condition but also elevates photosynthetic capacity and antioxidant enzyme activity [29,30,66]. Li et al. [63] found the role of melatonin in fortifying drought resistance through an uplifted antioxidant system and regulated abscisic acid metabolism. Furthermore, Ahmad et al. [67] reported that melatonin improved salinity stress tolerance by enhancing the antioxidant enzyme activities and photosynthetic capacity of maize seedlings. A significant enhancement in the quantum yield of photochemistry (qP) and the efficiency of photosystem II (ΦPSII) under salinity stress were identified with 100 μM melatonin application, a finding reported by Chen et al. [68], whereas a 20 μM melatonin application showed no significant effect on photochemical efficiency (Figure 3).

Melatonin also harmonizes carbon and N metabolisms to foster plant growth [69], highlighting the resistance to lead toxicity through the modulation of endogenous nitric oxide production [70] and bolstering photosynthesis and drought tolerance by controlling the ascorbate–glutathione cycle [71]. Additionally, melatonin regulates ABA metabolism that contributes to stomatal functionality under drought stress [63]. Despite no significant effect in control comparisons (no stress), melatonin significantly increased stomatal aperture and stomatal width under drought by 197.36 and 8.04%, respectively (Figure S1). Drought-induced stomatal closure led to significant reductions in stomatal length (82.3%), width (76.7%), aperture (72.3%), and density (79.1%), as shown by Zhao et al. [72], while melatonin treatment resulted in an 11.9% increase in stomatal length and a 12.0% increase in width. Moreover, melatonin-treated plants displayed a 1.3-fold larger stomatal aperture compared to those under salinity stress (Figure S1).

Under Cd stress, melatonin and N applications improved the physiological and biochemical features of maize during early development stages [31]. These treatments not only improved salinity tolerance, germination rates, and overall growth but also bolstered the antioxidant defense system and photosynthetic efficiency [67,68]. Alharby and Fahad [73] further reported that melatonin increased biochar efficiency, thereby fortifying maize’s drought tolerance through alteration in physiological and biochemical pathways. Additionally, melatonin was found to ameliorate maize’s response to abiotic stress by promoting growth, enhancing photosynthetic efficiency, regulating ABA metabolism, and strengthening the antioxidant defense mechanism.

### 2.2. Wheat

**Figure 4:**
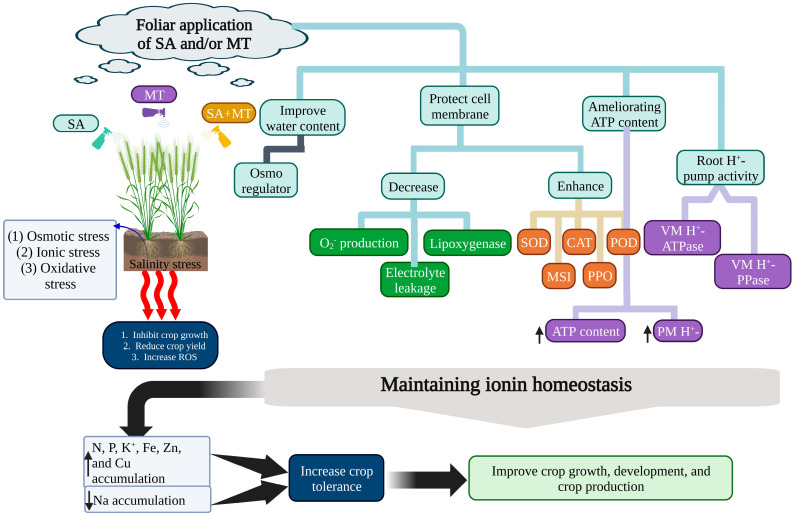
Foliar application of salicylic acid (SA) and melatonin (MT) reduces salinity stress in wheat by increasing root H^+^-pump activity, membrane stability index (MSI), polyphenol oxidase (PPO), ATP content, vacuole membrane H^+^-pump activity (VM H^+^), superoxide dismutase (SOD), catalase (CAT), peroxidase (POD), and water content and mitigating production of reactive oxygen species (ROS), resulting in preserving ionic homeostasis. The upward arrow indicates upregulation, and the downward arrow indicates downregulation.

Exogenous melatonin had a positive effect on wheat growth and stress tolerance under various stress conditions such as temperature stress, water stress, salinity stress, drought stress, and heavy metal stress. Studies showed that melatonin increases chlorophyll content and mitigates salinity stress in wheat [35], reduces boron toxicity [74], and diminishes heat stress effects by modulating the antioxidant defense system [75]. Talaat and Shawky [76] reported that combined applications of salicylic acid (SA) and melatonin regulate ion absorption and enhance selective substances’ accumulation in plant parts and membrane integrity, reducing oxidative damage and ROS production, thereby bolstering antioxidant enzyme activity, salt tolerance, and mineral nutrition (Figure 4).

Furthermore, melatonin coupled with zinc oxide nanoparticles (ZnONPs) improved wheat growth and zinc uptake under Cd stress [40], while chitosan–melatonin treatment enhanced growth and reduced heavy metal uptake in wheat grown on wastewater-polluted soil [37]. Cui et al. [50] and Cui et al. [77] reported melatonin’s benefits in overcoming drought and improving energy metabolism and autophagy under osmotic stress.

Moreover, Chen et al. [78] identified key genes in the melatonin biosynthesis pathway through a metabolite-based genome-wide association study, highlighting the importance of *TDC*, *T5H*, and one *SNAT* gene in the melatonin pathway’s metabolite accumulation. Increasing ZnONPs with melatonin application not only reduced grain Cd levels but also increased Zn content under Cd stress (Figure S2), with a notable increase in chlorophyll and carotenoid contents compared to the sole application of melatonin or ZnONPs. As compared to the control, chlorophyll a (63.5%) and b (103.8%) and carotenoid (153.3%) contents were significantly higher in plants treated with 100 µM melatonin + 100 mg kg^−1^ ZnONPs [40]. Melatonin application not only enhances growth and chlorophyll content but also ameliorates the adverse effect of various abiotic stresses, thereby improving nutrient uptake and cereal crops’ resilience [29,35,40,75,77].

### 2.3. Barley

Barley, the fourth-largest cereal crop globally, is widely cultivated across varied environments, including the challenging high altitudes of the Himalayan foothills, due to its remarkable adaptability [79]. Research has extensively explored melatonin’s impact on barley, particularly under stress conditions [60,80,81,82]. Melatonin exhibits a protective effect against temperature stress, resulting in improved crop growth and an increased expression of circadian clock genes in hulless barley seedlings under cold stress conditions [83]. It appears to modulate circadian gene expression rhythms, contributing to improved cold stress resilience. Furthermore, melatonin has increased cold tolerance in both wild-type and ABA-deficient mutant barley, alongside enhancing physiological parameters and gene expression related to wax biosynthesis under drought stress conditions [80,81].

Salinity stress impacts barley growth negatively, yet melatonin application has been found to mitigate these adverse effects by modulating the defense mechanism. Treatments with melatonin, calcium chloride (CaCl_2_), or their combination have significantly increased barley sprout growth under salinity stress (Figure S3A–C), as evidenced by the decreased fluorescence intensity and distributions of blue (O_2_^−^) and red (H_2_O_2_) signals in treated sprout, indicating enhanced cell membrane protection (Figure S3D,E). In contrast, LaCl_3_ and EGTA treatments increased ROS accumulation in NaCl–melatonin-treated sprouts, highlighting the protective role of melatonin [84]. Additionally, melatonin–Ca^2+^ has been suggested to boost phenolic acid accumulation and growth (Figure S3F). In the rhizosphere, melatonin modulates nitrogen-cycling microorganisms, increasing barley’s low-temperature tolerance [60].

Melatonin also reprograms rhizosphere microbial populations, enhancing barley’s resistance to low temperatures and heavy metal stress. Studies found that exogenous melatonin reduces polymetallic stress toxicity in barley, by modulating circadian genes, regulating rhizosphere microbial communities, and boosting antioxidant activity, serving as key defensive mechanisms [60,82,83,85]. These findings suggest melatonin’s potential to increase barley yields under abiotic stress. Furthermore, melatonin treatments reduce polymetallic stress toxicity [85], promote growth under cold stress [83], and affect endogenous melatonin levels in barley roots [80]. Melatonin’s protective effects extend to delaying leaf aging and preventing chlorophyll degradation, thereby enhancing barley’s resistance to various abiotic stresses and fortifying plant defense mechanisms [80].

### 2.4. Rice

Melatonin has been shown to alleviate multiple stressors in rice crops, such as temperature stress, water stress, salinity stress, drought stress, and heavy metal stress, because of its crucial role in regulating these processes. Its application to rice during its reproductive phase decreased heat stress [86], which improved the seed set, pollen viability, and yield [87,88]. In addition, melatonin increases resistance to *Xanthomonas oryzae* pv. oryzae, the causative agent of leaf blight in rice [89].

Melatonin treatment not only fosters growth and arsenic (As) tolerance due to promoting As accumulation and decreasing oxidative stress [90] but also elevates antioxidant enzyme activities in response to As exposure. This includes a significant increase in APX, CAT, POD, and SOD activities by 37, 26, 319, and 30%, in melatonin + As-treated plants and by 17, 31, 139, and 29% in anthocyanin + As-treated plants, respectively [91]. Moreover, melatonin boosts rice’s antioxidant defenses against salinity stress [92] and improves the antioxidant defense in sorghum seedlings under drought condition [93].

Exogenous gibberellin has been found to stimulate melatonin production, promoting melatonin-enriched rice growth. This hormonal interplay also extends to brassinosteroid, which acts as an endogenous melatonin inducer in rice seedlings [94], contributing to enhanced drought resistance and controlled water losses through stomatal regulation [95]. Furthermore, melatonin treatment was found to increase root and shoot dry weight under salinity stress [96]. Moreover, transgenic rice seedlings expressing rice tryptophan decarboxylase exhibited increased heavy metal tolerance when melatonin production was upregulated [97].

Melatonin has the potential to shield rice from abiotic stress, and its biosynthesis is regulated by enzymes like SNAT and ASMT [98]. Both light and dark conditions increased melatonin levels (Figure S4). Beyond abiotic stress, melatonin bolsters rice’s defense against bacterial pathogens by triggering immune system responses. The synergy between melatonin and growth regulators like gibberellin and brassinosteroid underscores the hormone’s integral role in enhancing rice’s resilience to environmental stresses [39,94,99,100].

### 2.5. Millet

Millet is an important cereal crop in dry and semiarid regions, but it is vulnerable to a variety of environmental stresses that can stunt its growth and reduce its yield. Melatonin has shown potential in reducing the negative effects of these stresses on crops. Melatonin increases the tolerance of millet crops to temperature stress by controlling the expression of genes in the thermotolerance pathway [55]. Similarly, water deficiency significantly limits the growth and productivity of millet, thereby reducing its overall yield. Melatonin specifically aids in optimizing water use efficiency by controlling the transpiration rate and the stomatal conductance which is critically adapted to the arid and semiarid conditions in which millets are commonly grown [101]. Previous studies have demonstrated that melatonin application improves millet’s tolerance to heavy metals and salinity stress by controlling the gene expression involved in pathways associated with the salinity stress response [55]. Furthermore, melatonin alleviates oxidative stress and increases drought stress resistance through the ABA-dependent signaling pathway, with varying concentrations yielding differential improvements in growth, physiological, and biochemical traits under drought stress. Notably, it increases proline content, indicative of enhanced drought tolerance [4]. Millet’s significance extends beyond its adaptability to water scarcity, positioning it as an essential cereal and bioenergy crop in regions including Asia and Europe [102]. As a pioneer crop, millet exhibits exceptional characteristics, including genetic diversity, a short life cycle, minimal water requirements, and robust tolerance to abiotic stress [4,103].

### 2.6. Sorghum

Sorghum is a common cereal crop that grows well in arid and semiarid regions because it is naturally resistant to several environmental stresses, including high temperatures, low humidity, high salinity, water scarcity, and nutrient shortages, but these stresses can still have a detrimental effect on crop production. Melatonin’s role in sorghum extends beyond stress mitigation; it enhances antioxidant metabolism which is crucial for maintaining photosynthetic efficiency under drought stress conditions [104]. Through a variety of mechanisms, melatonin increased the sorghum resistance to drought, including its capacity to promote seed germination and accelerate plant growth [105]. Additionally, melatonin minimizes oxidative stress and increases antioxidant defense systems, which mitigate the negative effects of drought stress in sorghum [93]. Melatonin coupled with arbuscular mycorrhizal fungi significantly increased antioxidant enzyme activities and alleviated drought stress in tobacco seedlings [53].

Moreover, the increase in plant growth and development from melatonin might be attributed to lower ROS production, higher antioxidant metabolism, and greater photosynthetic pigments in sorghum [104]. Koo and Arimura [106] revealed that melatonin upregulates the gene expression of sorghum crops associated with a biochemical defense system, leading to reduced ROS accumulation. Additionally, arbuscular mycorrhizal fungi coupled with melatonin alleviate drought stress and enhance plant growth [53]. The interlinked biosynthesis pathways of melatonin and serotonin underscore their roles in regulating plant stress response and development [23]. Genome-wide studies identifying genes involved in melatonin and serotonin biosynthesis confirm the crucial role of these hormones in stress response and development in various plant species [23].

## 3. Melatonin: Boosting Resilience in Cereal Crops

Melatonin application significantly increases crop production and quality, especially under stress conditions. The synergistic use of melatonin with N fertilization has been demonstrated to positively influence early growth, development, and related physiochemical attributes under Cd stress [31]. In addition, melatonin has been found to alleviate drought stress in maize by increasing hydraulic conductivity, which further underscores its utility in agricultural practices [71]. Notably, the application of melatonin improved sorghum seed germination under drought stress conditions, highlighting its broad applicability across different cereal crops [105]. However, the effects of melatonin on cereal crops are not uniform and can be influenced by various factors, including environmental stresses and nutrient homeostasis [55]. The potential of melatonin to improve plant growth and physiological attributes highlights the importance of continuous research in arid and semiarid regions. This continuous exploration is vital for optimizing melatonin application rates and methods, ensuring that cereal crops can achieve maximum resilience and productivity in the face of increasingly variable environmental conditions.

### 3.1. Molecular Insights into Melatonin-Mediated Plant Stress Tolerance

The application of melatonin across different plant species modulates specific genes and biochemical pathways, enhancing stress tolerance (Table 1). In maize, melatonin treatment leads to the upregulation of genes involved in N assimilation, carbohydrate metabolism, and photosynthesis, while genes involved in sugar metabolism are downregulated, collectively contributing to improve drought tolerance [107]. In barley, melatonin increases the expression of waxy genes, regulating leaf stomatal behavior, as well as carbon and nitrogen metabolism, which in turn affects related gene expression [51,72]. Furthermore, melatonin induces the expression of genes involved in flavonoid synthesis and certain transcription factors, thereby enhancing plants’ drought tolerance by altering plant hormone signaling [108]. In wheat, melatonin augments antioxidant enzyme activities during germination and alters phytohormonal responses to salinity, thereby improving salt stress tolerance [109]. Transcriptome analyses have identified flavonoid production and antioxidant responses as key mechanisms by which melatonin enhances wheat’s drought resistance [110]. In rice, melatonin application during seed germination positively affects metabolic pathways, leading to increased salt tolerance, and demonstrates a capacity to delay leaf senescence, indicating a broad impact on key physiological processes [111]. Moreover, under cold and salt stress, melatonin restored circadian rhythms in barley and decreased the presence of stress markers, thus boosting plant growth [83]. These findings highlight the role of melatonin in regulating important genetic and biological functions that improve stress tolerance and plant growth in cereal crops (Table 1).

### 3.2. Melatonin’s Role in Enhancing Photosynthesis, Nutrient Uptake, and Seed Development

Exogenous melatonin application significantly enhances the photosynthetic capacity of plants, particularly under stress conditions like drought and salinity stress [29,68]. It increases the hydraulic conductivity in maize during drought stress, simulated with polyethylene glycol (PEG), facilitating the maintenance of optimal transpiration and photosynthetic rates [63]. Melatonin also improves growth attributes and antioxidant enzyme activities, effectively reducing ROS accumulations in maize seedlings under drought stress [29]. The combined application of melatonin and N modulates the growth, physiological, and biochemical traits in maize under Cd stress [40] and significantly bolsters drought tolerance and overall growth in sweet sorghum [105]. Furthermore, melatonin positively influences the nutrient uptake, thereby enhancing growth and physiological characteristics in maize and sorghum and augmenting their resilience to drought and Cd stresses [31,71,105]. The improvement in the hydraulic conductance in maize, attributed to melatonin, increases tolerance to temporary water deficits, reinforcing seedlings’ photosynthetic capabilities and antioxidant defense during salt stress [68]. The genome-wide identification of genes associated with melatonin biosynthesis in plants underscores its conserved function in stress response and development, highlighting the potential of melatonin in promoting plant growth and nutrient assimilation under a variety of stress conditions [23].

Melatonin regulates key physiological and biochemical processes crucial for flowering and seed development. Its application improves maize growth, photosynthesis, and the antioxidant defense system under drought stress conditions [15] and assists in maintaining nutrient homeostasis and managing stress signaling pathways in plants. The complex interplay between plant stress responses and signaling pathways elucidates the intricate effects of melatonin on flowering and seed formation. Melatonin’s role becomes particularly crucial under stress conditions associated with flowering and seed formation, also affecting water intake and transportation [30,35,94]. A comprehensive analysis of genes involved in serotonin and melatonin biosynthesis clarifies the molecular mechanisms underlying melatonin’s impact on these processes [23], shedding light on the diverse ways melatonin supports plant growth and development under abiotic stress.

## 4. Enhancing Crop Resilience and Sustainability in Agriculture

Melatonin, initially recognized for its role in regulating the circadian rhythm in animals, has gained considerable attention in agricultural research for its potential in sustainable agriculture. Extensive studies have demonstrated melatonin’s effectiveness in enhancing stress tolerance in cereal corps including maize, under conditions such as drought, by increasing hydraulic conductance and improving growth, photosynthetic capacity, and antioxidant defenses [29,30]. These findings highlight melatonin’s importance for cereal farming’s long-term sustainability [101,106]. Despite these promising results, further exploration is required to fully understand melatonin’s long-term impacts and optimize application methods.

Melatonin is a suitable choice for agricultural applications, owing to its biodegradable nature and low toxicity [15], presenting a safe and eco-friendly option for enhancing crop growth and stress tolerance, particularly in challenging environments [63,92,124]. Its rapid degradation in soil without causing toxicity, even at high concentrations, positions melatonin as a sustainable growth regulator that aligns with environmentally responsible farming practices [16,125,126]. Melatonin promotes crop resilience and productivity by regulating gene expression and promoting antioxidant activity, which is consistent with sustainable farming practices.

However, the sustainability of melatonin use in agriculture depends on several factors, including the environmental impact of its production and extraction processes. The long-term effects on soil health, biodiversity, and ecosystem function require thorough investigation to ensure that melatonin use does not lead to ecological disruptions or pest and pathogen resistance [26,127]. Comprehensive studies covering environmental, economic, and social aspects are crucial to assess melatonin’s viability as an agricultural growth regulator.

This review has highlighted melatonin’s potential in improving crop growth and stress tolerance, particularly in cereal crops, suggesting that melatonin application enhances plant resilience to environmental stresses like drought and Cd toxicity by positively affecting plant growth, photosynthetic capacity, and antioxidant defense systems [71,123]. Its synergistic effects with arbuscular mycorrhizal fungi, for example, enhance plant growth and drought stress tolerance, underscoring the importance of understanding the underlying molecular mechanisms [53]. The exploration of genes responsible for melatonin biosynthesis and the studies of signaling pathways and nutrient homeostasis under stress conditions could further improve sustainable cereal production.

Despite the promising potential of melatonin in mitigating environmental stress on crop growth, challenges remain. Further research should aim for a comprehensive understanding of melatonin’s signaling pathways, discover new receptors, and develop more environmentally friendly application methods. The exploration of melatonin’s synergistic effects with other compounds, its impact on plant–microbe interactions, and its role in plant defense against biotic stresses are vital areas for further research. Understanding melatonin’s interactions with other plant hormones and elucidating its biosynthesis in crops could lead to the development of stress-tolerant cultivars with enhanced yields and nutritional quality, thereby contributing to innovative and sustainable agricultural practices.

This review underscores the significant impact of melatonin on plant stress response mechanisms and its potential applications in enhancing crop production and environmental management. By leveraging the molecular insights from these studies, strategies to mitigate stress impacts on plant development and improve agriculture sustainability can be developed. Further research is essential to fully understand melatonin’s effects on crop yield and quality under various abiotic stress conditions, paving the way for innovative approaches that bolster agricultural resilience.

## Figures and Tables

**Table 1:** Melatonin-induced molecular responses in cereal crops under stress conditions. Regulatory mechanisms of genes in melatonin signaling pathways for regulating abiotic stress tolerance, as well as cereal crops’ growth.

Species	Latin Name	Stress Types	Upregulated Genes/Metabolic Pathways	Downregulated Genes/Metabolic Pathways	Major Findings	References
Maize	*Zea Mays*	Drought stress	LHC, Psb, PRK, Rubisco, GAPDH, SPS, AGP, SBE, GS, NR, PetE, beta	INV, SuSy, AMY, BMY, GDH, AMT	Enhances drought tolerance in maize by protecting photosynthetic efficiency, promoting carbohydrate and N metabolism, and coordinating carbon and N assimilation, ultimately supporting plant growth and stress resilience.	[107]
			PAL, C4H, 4CL, HCT, CHS, CHI, F3′5′H, DFR, ERFs, NACs, MYBs, bHLHs, ERF4, ERF81, ERF110	---	Melatonin application during drought stress leads to the upregulation of genes associated with flavonoid synthesis in roots, activation of specific transcription factors, and modulation of plant hormone signaling pathways, resulting in increased flavonoid accumulation and improved drought tolerance.	[108]
			AUX1, AUX/IAA, SAUR, GID2, GID1, ABF, SIMKK, ERF1/2, BAK1, JAZ, TGA, GST, pepA, CNGCs, CDPKs, CaM/CMLs, DELLA, MYC2	GH3, IF, EIN3, GPX, APX, PIF4, B-ARR, EIN3	Exogenous melatonin in maize seedlings under drought stress conditions leads to increased drought tolerance by promoting growth, enhancing antioxidant defenses, modulating calcium signaling and transcription factors, and regulating the plant hormone signaling network, including jasmonic acid biosynthesis and signaling pathways.	[112]
			*Zmsps1*, *ZmPEPC*, *ZmrbcS*, *ZmrbcL*, SuSy, AGPas, PEPC	GDH	Melatonin alleviates the negative impacts of drought stress on maize by enhancing photosynthesis, promoting stomatal opening, and modulating carbon and N metabolism.	[72]
			IVR2, SUS2, CWI/VI, SUS, SPS	dINV, SUS1, INVINH	Melatonin can either promote or inhibit maize seedling growth, with its concentration-dependent effects on sugar metabolism and carbohydrate partitioning genes leading to alterations in photosynthesis, hexose accumulation, and sucrose phloem loading, providing novel insights into the regulation of plant growth.	[113]
			*ABA8ox1b*, *ABA8ox3a*, *ABA8ox3b*, *NCED1*	*ABA8ox1a*	Melatonin pre-treatment in maize seedlings mitigates the adverse effects of drought stress by maintaining leaf water content, enhancing antioxidant systems, reducing ROS accumulation, preventing chlorophyll degradation, promoting stomatal reopening, and regulating ABA levels, ultimately leading to improved drought tolerance and photosynthesis	[63]
			*ZmPIP1;2*, *ZmPIP2;2*, *ZmPIP1;5*, *ZmPIP2;5*	---	Melatonin treatment in maize seedlings subjected to water deficiency results in increased aquaporin activity, improved root hydraulic conductance, higher leaf water potential, and enhanced tolerance to drought stress, all of which contribute to improved water uptake and transport.	[30]
		Aluminum	LHC, Psb, Psa, Pet, gamma, delta, Rubisco, PGK, GAPDH, FBP, PRK, SPS, AGP, GBSS, SS, SBE, TPS, TPP, NRT, NR, GS, GOGAT,	AMY, BMY, SuSy, CWINV, GDH, AMT	Melatonin application mitigates aluminum-induced growth inhibition in maize by enhancing photosynthetic efficiency, improving carbon and N metabolism, and reducing oxidative stress, thereby highlighting its potential as an eco-friendly strategy for sustainable crop production in acidic soils.	[114]
		Chromium	UGDH, GAE, GAUT, CSL, XYL, PME, GST, PCS, SOD, CAT, POD, GR, APX	RBOH, PAO	Melatonin plays a critical role by modulating osmotic balance, bolstering the plant’s antioxidant defense systems, and sustaining photosynthetic activity and mitigating cadmium toxicity in plants through its role in regulating metal transporters and antioxidant systems, and the revelation with melatonin enhances the binding capacity of cell walls in maize by influencing the biosynthesis of pectin and hemicellulose.	[115]
Wheat	*Triticum aestivum* L.	Salt stress	Oxalate oxidase activity, glutathione transferase activity, oxidoreductase activity, and establishment	Sodium ion import across plasma membrane, potassium ion transmembrane transporter activity, cellular chemical homeostasis, cellular lipid catabolic process, and hormone catabolic process	Melatonin enhances wheat seed germination by increasing antioxidant enzyme activities, modifying phytohormone responses, regulating ion transport pathways, and influencing the synthesis of protective substances such as flavonoids, ultimately improving salt tolerance during germination.	[109]
		Drought stress	NCED, PP2C, SnRK2, ARF, ARG, ODC1, ROCD, ARD, PRDX6, HK	crtZ, PYR/PYL, AROK, SAUR, CD13, ASO, NADH, scrK, PFP, ALDO	Drought stress significantly impacts wheat production and quality; several genes related to wheat drought tolerance have been identified through transcriptome analysis that revealed key tolerance mechanisms involved in flavonoid biosynthesis, plant hormone signaling, phenolamide production, and antioxidant responses.	[110]
			GAS, C4H, CHS,	CHI, RAFS, STS, FRSs, SUS	Significant improvement in drought tolerance in wheat seedlings through exogenous melatonin, as evidenced by enhanced physiological parameters, transcriptomic and metabolomic analyses revealing the key pathways involved in drought response, and the identification of potential molecular mechanisms related to flavonoid biosynthesis and carbohydrate metabolism.	[116]
		Agrobacterium-tumefaciens	PFK, gapN, gpmI, pyk, RAFS, tktA, IDH1, GLT1, POP2	PDHA, PDHB, DLAT, DLD, LSC1, LSC2, ACO, fumC, MDH2, GSS, SDHA	The identification of the key pathways and genes involved in the response of immature wheat embryos to Agrobacterium infection, highlighting the activation of energy and stress-related pathways, changes in redox substances, and the complex regulatory network.	[117]
Rice	*Oryza sativa*	Salt stress	PAL, GA2, RCI3, PRX4, PRX6, PRX10, bHLH TFs	NECD	Exogenous melatonin treatment enhances rice seed germination under salt stress by promoting antioxidant activity, modulating metabolic pathways, and influencing phytohormone concentrations, suggesting its potential use in improving salt tolerance in rice.	[111]
			TDC, T5H, ASMT, T6PP, TRE, GoIS, HSF,	GRAS, WRKY, PLATZ	Salt stress negatively impacts the growth and seed quality of rice plants, leading to changes in gene expression, mineral accumulation, and the upregulation of various metabolic pathways and transcription factors in developing rice seeds.	[118]
			*OsEXPB2*, Hsp40, TFs, auxin, ABA	*OsBBX20*, *OsLTP2.12*,	Exogenous melatonin in rice seedlings leads to the upregulation of specific transcription factors, activation of phytohormone signaling pathways, and modulation of metabolite profiles, collectively contributing to enhanced salt tolerance and improved stress responses.	[119]
			Tify, TRAF, SRS, RWP-PK, mTTERF, HMG, GRAS, C2C2-YABBY, C2C2-CO,	bZIP, NAC, TFs, GRAS, mTERF, Tify, HSF, MYB, WRKY	Melatonin delays leaf senescence and cell death in rice by enhancing oxidative stress tolerance, reducing hydrogen peroxide (H_2_O_2_) accumulation, and modulating gene expression and the antioxidant defense system, thus extending the longevity of leaves and improving stress resistance.	[120]
Barly	*Hordeum vulgare*	Cold stress	*HvCCA1*, *HvPRR73*, *HvELF3*	*HvTOC1*, *HvPRR59*, *HvPRR95*, *HvLUX*, *HvGI*	A total of 1 µM of exogenous melatonin restores the rhythmicity of circadian clock genes, enhances the accumulation of photosynthetic pigments, and reduces stress-related indicators, ultimately promoting plant growth under cold stress conditions in hulless barley.	[83]
			HvSOD1, HvCAT2,	---	Melatonin can re-establish circadian rhythms in H_2_O_2_ levels, antioxidant enzyme activities (SOD and CAT), and the PRX-SO2/3 rhythmic marker under cold stress conditions in hulless barley seedlings. Additionally, melatonin influences the circadian rhythmicity of MDA and soluble sugars.	[82]
		Salt stress	4CL, APL, C4H,	F5H	Melatonin significantly increases the levels of phenolic acids, including ferulic acid, p-coumaric acid, and p-hydroxybenzoic acid, while reducing oxidative damage, enhancing biomass, and promoting sprout growth of barley under salt stress.	[121]
			TDC, T5H, F2CTV7 Osmotin/thaumatin-like_sf, A0A287WVK2 Tryptophan synthase, F2E7G3 methyltransferase activity, AOA287M228 delta-1-pyrroline-5-carboxylate synthase	F2D9A0 α/β hydrolase	Melatonin significantly improves salt stress tolerance in germinating hulless barley seeds, as indicated by the increased germination rate and root length and the reduced oxidative stress levels, with an underlying influence on multiple molecular and metabolic pathways related to microtubule-associated proteins, motor proteins, histone H2B, energy metabolism, amino acid metabolism, ion transport, antioxidant defenses, and vacuolar ion exchange.	[122]
Millet	*Panicum miliaceum* L.	Drought stress	SNAT, TDC, AUX/IAA, ABF, AUX1,	PP2Cs, MPK6, ChlH, ChlI, and ChlD	Drought-tolerant and drought-sensitive broomcorn millet varieties (DT 43 and DS 190) show the differential responses of these varieties to drought stress, the role of plant hormone signaling and MAPK pathways in conferring drought resistance to DT 43, the impact of carbon and N metabolism on senescence under drought stress, and the beneficial effects of melatonin treatment in enhancing drought resistance by improving photosynthetic and antioxidant capacities while mitigating transcriptional responses in both varieties.	[4]
		Cadmium	superoxide dismutase SOD-[Fe] 2, Fe superoxide dismutase, Peroxiredoxin 2C	---	Melatonin application through soil and foliar spray significantly reduces Cd accumulation, mitigates oxidative stress, improves growth parameters, enhances the expression of antioxidant-related genes, and increases Cd stress tolerance in pearl millet.	[123]

The table includes data from multiple studies that investigated the molecular responses of diverse plant species to melatonin treatment under stress conditions. Upregulated and downregulated genes, as well as key discoveries, are reported for each species.

## Supplementary Materials

The supporting information can be downloaded at https://www.mdpi.com/article/10.3390/ijms25084551/s1.

## References

[1] Kang X., Gao W., Cui B., Abd El-Aty A. (2023). Structure and genetic regulation of starch formation in sorghum (*Sorghum bicolor* (L.) Moench) endosperm: A review. Int. J. Biol. Macromol..

[2] Miao H., Li D., Wang J., Sun Y., Liu L., Liu R., Li H. (2020). Effects of melatonin on the growth and yield of wheat under drought condition. Agric. Res. Arid Areas.

[3] Kaul J., Jain K., Olakh D. (2019). An overview on role of yellow maize in food, feed and nutrition security. Int. J. Curr. Microbiol. Appl. Sci..

[4] Yuan Y., Liu L., Gao Y., Yang Q., Dong K., Liu T., Feng B. (2022). Comparative analysis of drought-responsive physiological and transcriptome in broomcorn millet (*Panicum miliaceum* L.) genotypes with contrasting drought tolerance. Ind. Crops Prod..

[5] Ostmeyer T.J., Bahuguna R.N., Kirkham M., Bean S., Jagadish S. (2022). Enhancing sorghum yield through efficient use of nitrogen–challenges and opportunities. Front. Plant Sci..

[6] Muhammad I., Khan A., Mustafa A.-Z., Elshikh M.S., Shen W. (2024). Elucidating the modulatory effect of melatonin on enzyme activity and oxidative stress in wheat: A global meta-analysis. Physiol. Plant..

[7] Shiferaw B., Prasanna B.M., Hellin J., Bänziger M. (2011). Crops that feed the world 6. Past successes and future challenges to the role played by maize in global food security. Food Secur..

[8] Rai P.K., Song H., Kim K.-H. (2023). Nanoparticles modulate heavy-metal and arsenic stress in food crops: Hormesis for food security/safety and public health. Sci. Total Environ..

[9] Vancov T., McIntosh S. (2011). Alkali pretreatment of cereal crop residues for second-generation biofuels. Energy Fuels.

[10] Onyeonagu C., Njoku O. (2010). Crop residues and agro-industrial by-products used in traditional sheep and goat production in rural communities of Markudi LGA. Agro-Science.

[11] Devi S., Gupta C., Jat S.L., Parmar M. (2017). Crop residue recycling for economic and environmental sustainability: The case of India. Open Agric..

[12] Yonar A., Yonar H., Mishra P., Kumari B., Abotaleb M., Badr A. (2021). Modeling and forecasting of wheat of South Asian region countries and role in food security. Adv. Comput. Intell..

[13] Subudhi P.K. (2023). Molecular Research in Rice. Int. J. Mol. Sci..

[14] Yang Z., Xie C., Bao Y., Liu F., Wang H., Wang Y. (2023). Oat: Current state and challenges in plant-based food applications. Trends Food Sci. Technol..

[15] Muhammad I., Yang L., Ahmad S., Mosaad I.S.M., Al-Ghamdi A.A., Abbasi A.M., Zhou X.-B. (2022). Melatonin Application Alleviates Stress-Induced Photosynthetic Inhibition and Oxidative Damage by Regulating Antioxidant Defense System of Maize: A Meta-Analysis. Antioxidants.

[16] Kosakivska I.V., Vedenicheva N.P., Babenko L.M., Voytenko L.V., Romanenko K.O., Vasyuk V.A. (2022). Exogenous phytohormones in the regulation of growth and development of cereals under abiotic stresses. Mol. Biol. Rep..

[17] Shen J., Qin C., Qin Y., Du M., Begum N., Lian H. (2023). Acetylcholine Alleviates Salt Stress in Zea mays L. by Promoting Seed Germination and Regulating Phytohormone Level and Antioxidant Capacity. J. Plant Growth Regul..

[18] Abdollahi M., Ranjbar A., Shadnia S., Nikfar S., Rezaie A. (2004). Pesticides and oxidative stress: A review. Med. Sci. Monit..

[19] Iwaniuk P., Konecki R., Kaczynski P., Rysbekova A., Lozowicka B. (2022). Influence of seven levels of chemical/biostimulator protection on amino acid profile and yield traits in wheat. Crop J..

[20] Weller S., Culbreath A., Gianessi L., Godfrey L., Jachetta J., Norsworthy J., Palumbo J., Madsen J. (2014). The Contributions of Pesticides to Pest Management in Meeting the Global Need for Food Production by 2050.

[21] Wang J., Zhang L., Tao N., Wang X., Deng S., Li M., Zu Y., Xu C. (2023). Small Peptides Isolated from Enzymatic Hydrolyzate of Pneumatophorus japonicus Bone Promote Sleep by Regulating Circadian Rhythms. Foods.

[22] Pan Y., Xu X., Li L., Sun Q., Wang Q., Huang H., Tong Z., Zhang J. (2023). Melatonin-mediated development and abiotic stress tolerance in plants. Front. Plant Sci..

[23] Bhowal B., Bhattacharjee A., Goswami K., Sanan-Mishra N., Singla-Pareek S.L., Kaur C., Sopory S. (2021). Serotonin and Melatonin Biosynthesis in Plants: Genome-Wide Identification of the Genes and Their Expression Reveal a Conserved Role in Stress and Development. Int. J. Mol. Sci..

[24] Lei K., Sun S., Zhong K., Li S., Hu H., Sun C., Zheng Q., Tian Z., Dai T., Sun J. (2021). Seed soaking with melatonin promotes seed germination under chromium stress via enhancing reserve mobilization and antioxidant metabolism in wheat. Ecotoxicol. Environ. Saf..

[25] Ma S., Gai P., Geng B., Wang Y., Ullah N., Zhang W., Zhang H., Fan Y., Huang Z. (2022). Exogenous Melatonin Improves Waterlogging Tolerance in Wheat through Promoting Antioxidant Enzymatic Activity and Carbon Assimilation. Agronomy.

[26] Zhang N., Zhao B., Zhang H.J., Weeda S., Yang C., Yang Z.C., Ren S., Guo Y.D. (2013). Melatonin promotes water-stress tolerance, lateral root formation, and seed germination in cucumber (*Cucumis sativus* L.). J. Pineal Res..

[27] Liu G., Hu Q., Zhang X., Jiang J., Zhang Y., Zhang Z. (2022). Melatonin biosynthesis and signal transduction in plants in response to environmental conditions. J. Exp. Bot..

[28] Arnao M., Hernández-Ruiz J. (2015). Melatonin: Synthesis from tryptophan and its role in higher plant. Amino Acids in Higher Plants.

[29] Ahmad S., Kamran M., Ding R., Meng X., Wang H., Ahmad I., Fahad S., Han Q. (2019). Exogenous melatonin confers drought stress by promoting plant growth, photosynthetic capacity and antioxidant defense system of maize seedlings. PeerJ.

[30] Qiao Y., Ren J., Yin L., Liu Y., Deng X., Liu P., Wang S. (2020). Exogenous melatonin alleviates PEG-induced short-term water deficiency in maize by increasing hydraulic conductance. BMC Plant Biol..

[31] Ma L., Huang Z., Li S., Ashraf U., Yang W., Liu H., Xu D., Li W., Mo Z. (2021). Melatonin and nitrogen applications modulate early growth and related physio-biochemical attributes in maize under Cd stress. J. Soil Sci. Plant Nutr..

[32] Ren J., Ye J., Yin L., Li G., Deng X., Wang S. (2020). Exogenous Melatonin Improves Salt Tolerance by Mitigating Osmotic, Ion, and Oxidative Stresses in Maize Seedlings. Agronomy.

[33] Malik Z., Afzal S., Dawood M., Abbasi G.H., Khan M.I., Kamran M., Zhran M., Hayat M.T., Aslam M.N., Rafay M. (2022). Exogenous melatonin mitigates chromium toxicity in maize seedlings by modulating antioxidant system and suppresses chromium uptake and oxidative stress. Environ. Geochem. Health.

[34] Raza A., Mubarik M.S., Sharif R., Habib M., Jabeen W., Zhang C., Chen H., Chen Z.H., Siddique K.H., Zhuang W. (2023). Developing drought-smart, ready-to-grow future crops. Plant Genome.

[35] Ahmad I., Munsif F., Mihoub A., Jamal A., Saeed M.F., Babar S., Fawad M., Zia A. (2022). Beneficial Effect of Melatonin on Growth and Chlorophyll Content in Wheat (*Triticum aestivum* L.) Grown Under Salt Stress Conditions. Gesunde Pflanz..

[36] Jiang D., Lu B., Liu L., Duan W., Meng Y., Li J., Zhang K., Sun H., Zhang Y., Dong H. (2021). Exogenous melatonin improves the salt tolerance of cotton by removing active oxygen and protecting photosynthetic organs. BMC Plant Biol..

[37] Dradrach A., Iqbal M., Lewinska K., Jedroszka N., Gull e.F., Rana M.A.K., Tanzeem-ul-Haq H.S. (2022). Effects of Soil Application of Chitosan and Foliar Melatonin on Growth, Photosynthesis, and Heavy Metals Accumulation in Wheat Growing on Wastewater Polluted Soil. Sustainability.

[38] Ou C., Cheng W., Wang Z., Yao X., Yang S. (2023). Exogenous melatonin enhances Cd stress tolerance in Platycladus orientalis seedlings by improving mineral nutrient uptake and oxidative stress. Ecotoxicol. Environ. Saf..

[39] Hwang O.J., Back K. (2022). Molecular Regulation of Antioxidant Melatonin Biosynthesis by Brassinosteroid Acting as an Endogenous Elicitor of Melatonin Induction in Rice Seedlings. Antioxidants.

[40] Chen F., Li Y., Zia-ur-Rehman M., Hussain S.M., Qayyum M.F., Rizwan M., Alharby H.F., Alabdallah N.M., Alharbi B.M., Ali S. (2023). Combined effects of zinc oxide nanoparticles and melatonin on wheat growth, chlorophyll contents, cadmium (Cd) and zinc uptake under Cd stress. Sci. Total Environ..

[41] Karami-Mohajeri S., Abdollahi M. (2011). Toxic influence of organophosphate, carbamate, and organochlorine pesticides on cellular metabolism of lipids, proteins, and carbohydrates: A systematic review. Hum. Exp. Toxicol..

[42] Asghari M.H., Moloudizargari M., Bahadar H., Abdollahi M. (2017). A review of the protective effect of melatonin in pesticide-induced toxicity. Expert Opin. Drug Metab. Toxicol..

[43] Iwaniuk P., Łuniewski S., Kaczyński P., Łozowicka B. (2023). The influence of humic acids and nitrophenols on metabolic compounds and pesticide behavior in wheat under biotic stress. Agronomy.

[44] Tiwari R.K., Lal M.K., Naga K.C., Kumar R., Chourasia K.N., Subhash S., Kumar D., Sharma S. (2020). Emerging roles of melatonin in mitigating abiotic and biotic stresses of horticultural crops. Sci. Hortic..

[45] Giraldo Acosta M., Cano A., Hernández-Ruiz J., Arnao M.B. (2022). Melatonin as a possible natural safener in crops. Plants.

[46] Sun C., Liu L., Wang L., Li B., Jin C., Lin X. (2021). Melatonin: A master regulator of plant development and stress responses. J. Integr. Plant Biol..

[47] Tiwari R.K., Lal M.K., Kumar R., Chourasia K.N., Naga K.C., Kumar D., Das S.K., Zinta G. (2021). Mechanistic insights on melatonin-mediated drought stress mitigation in plants. Physiol. Plant..

[48] Chen J., Qin H., Zhang B., Mao W., Lou L., Shen C., Mao J., Lin Q. (2022). Development of melatonin nano-delivery systems to reduce cadmium accumulation in rice (*Oryza sativa* L.) seedlings: Insights from photosynthetic efficiency, antioxidative response and gene expression. Environ. Exp. Bot..

[49] Sakouhi L., Kadri O., Werghi S., Massoud M.B., Kharbech O., Murata Y., Chaoui A. (2023). Seed pretreatment with melatonin confers cadmium tolerance to chickpea seedlings through cellular redox homeostasis and antioxidant gene expression improvement. Environ. Sci. Pollut. Res..

[50] Cui G., Zhao X., Liu S., Sun F., Zhang C., Xi Y. (2017). Beneficial effects of melatonin in overcoming drought stress in wheat seedlings. Plant Physiol. Biochem..

[51] Lu X., Xu L., Zhang L., Zhang W., Han J., Tong T., Zhang X., Xue D. (2020). Effects of exogenous melatonin on physiology and waxy genes expression in barley under drought stress. Plant Physiol. J..

[52] Lu X., Min W., Shi Y., Tian L., Li P., Ma T., Zhang Y., Luo C. (2022). Exogenous melatonin alleviates alkaline stress by removing reactive oxygen species and promoting antioxidant defence in rice seedlings. Front. Plant Sci..

[53] Liu L., Li D., Ma Y., Shen H., Zhao S., Wang Y. (2021). Combined Application of Arbuscular Mycorrhizal Fungi and Exogenous Melatonin Alleviates Drought Stress and Improves Plant Growth in Tobacco Seedlings. J. Plant Growth Regul..

[54] Wei H., He W., Kuang Y., Wang Z., Wang Y., Hu W., Tang M., Chen H. (2023). Arbuscular mycorrhizal symbiosis and melatonin synergistically suppress heat-induced leaf senescence involves in abscisic acid, gibberellin, and cytokinin-mediated pathways in perennial ryegrass. Environ. Exp. Bot..

[55] Banerjee A., Roychoudhury A. (2022). Explicating the cross-talks between nanoparticles, signaling pathways and nutrient homeostasis during environmental stresses and xenobiotic toxicity for sustainable cultivation of cereals. Chemosphere.

[56] Del Río L.A., Corpas F.J., López-Huertas E., Palma J.M. (2018). Plant superoxide dismutases: Function under abiotic stress conditions. Antioxidants and Antioxidant Enzymes in Higher Plants.

[57] Rodziewicz P., Swarcewicz B., Chmielewska K., Wojakowska A., Stobiecki M. (2014). Influence of abiotic stresses on plant proteome and metabolome changes. Acta Physiol. Plant..

[58] Zhang Z., Guo L., Sun H., Wu J., Liu L., Wang J., Wang B., Wang Q., Sun Z., Li D. (2023). Melatonin Increases Drought Resistance through Regulating the Fine Root and Root Hair Morphology of Wheat Revealed with RhizoPot. Agronomy.

[59] Sun Z., Li J., Guo D., Wang T., Tian Y., Ma C., Liu X., Wang C., Zheng X. (2023). Melatonin enhances KCl salinity tolerance by maintaining K+ homeostasis in Malus hupehensis. Plant Biotechnol. J..

[60] Jiang M., Ye F., Liu F., Brestic M., Li X. (2022). Rhizosphere melatonin application reprograms nitrogen-cycling related microorganisms to modulate low temperature response in barley. Front. Plant Sci..

[61] Gill S.S., Tuteja N. (2010). Reactive oxygen species and antioxidant machinery in abiotic stress tolerance in crop plants. Plant Physiol. Biochem..

[62] Rizwan M., Nawaz A., Irshad S., Manoharadas S. (2024). Exogenously applied melatonin enhanced chromium tolerance in pepper by up-regulating the photosynthetic apparatus and antioxidant machinery. Sci. Hortic..

[63] Li Z., Su X., Chen Y., Fan X., He L., Guo J., Wang Y., Yang Q. (2021). Melatonin Improves Drought Resistance in Maize Seedlings by Enhancing the Antioxidant System and Regulating Abscisic Acid Metabolism to Maintain Stomatal Opening Under PEG-Induced Drought. J. Plant Biol..

[64] Muhammad I., Yang L., Ahmad S., Farooq S., Khan A., Muhammad N., Ullah S., Adnan M., Ali S., Liang Q.P. (2023). Melatonin-priming enhances maize seedling drought tolerance by regulating the antioxidant defense system. Plant Physiol..

[65] Cao Q., Li G., Cui Z., Yang F., Jiang X., Diallo L., Kong F. (2019). Seed Priming with Melatonin Improves the Seed Germination of Waxy Maize under Chilling Stress via Promoting the Antioxidant System and Starch Metabolism. Sci. Rep..

[66] Huang B., Chen Y.-E., Zhao Y.-Q., Ding C.-B., Liao J.-Q., Hu C., Zhou L.-J., Zhang Z.-W., Yuan S., Yuan M. (2019). Exogenous Melatonin Alleviates Oxidative Damages and Protects Photosystem II in Maize Seedlings Under Drought Stress. Front. Plant Sci..

[67] Ahmad S., Cui W., Kamran M., Ahmad I., Meng X., Wu X., Su W., Javed T., El-Serehy H.A., Jia Z. (2021). Exogenous Application of Melatonin Induces Tolerance to Salt Stress by Improving the Photosynthetic Efficiency and Antioxidant Defense System of Maize Seedling. J. Plant Growth Regul..

[68] Chen Y.-E., Mao J.-J., Sun L.-Q., Huang B., Ding C.-B., Gu Y., Liao J.-Q., Hu C., Zhang Z.-W., Yuan S. (2018). Exogenous melatonin enhances salt stress tolerance in maize seedlings by improving antioxidant and photosynthetic capacity. Physiol. Plant..

[69] Erdal S. (2019). Melatonin promotes plant growth by maintaining integration and coordination between carbon and nitrogen metabolisms. Plant Cell Rep..

[70] Okant M., Kaya C. (2019). The role of endogenous nitric oxide in melatonin-improved tolerance to lead toxicity in maize plants. Environ. Sci. Pollut. Res..

[71] Guo Y.Y., Li H.J., Zhao C.F., Xue J.Q., Zhang R.H. (2020). Exogenous Melatonin Improves Drought Tolerance in Maize Seedlings by Regulating Photosynthesis and the Ascorbate-Glutathione Cycle. Russ. J. Plant Physiol..

[72] Zhao C., Guo H., Wang J., Wang Y., Zhang R. (2021). Melatonin Enhances Drought Tolerance by Regulating Leaf Stomatal Behavior, Carbon and Nitrogen Metabolism, and Related Gene Expression in Maize Plants. Front. Plant Sci..

[73] Alharby H.F., Fahad S. (2020). Melatonin application enhances biochar efficiency for drought tolerance in maize varieties: Modifications in physio-biochemical machinery. Agron. J..

[74] Al-Huqail A.A., Khan M.N., Ali H.M., Siddiqui M.H., Al-Huqail A.A., AlZuaibr F.M., Al-Muwayhi M.A., Marraiki N., Al-Humaid L.A. (2020). Exogenous melatonin mitigates boron toxicity in wheat. Ecotoxicol. Environ. Saf..

[75] Buttar Z.A., Wu S.N., Arnao M.B., Wang C., Ullah I., Wang C. (2020). Melatonin Suppressed the Heat Stress-Induced Damage in Wheat Seedlings by Modulating the Antioxidant Machinery. Plants.

[76] Talaat N.B., Shawky B.T. (2022). Synergistic Effects of Salicylic Acid and Melatonin on Modulating Ion Homeostasis in Salt-Stressed Wheat (*Triticum aestivum* L.) Plants by Enhancing Root H+-Pump Activity. Plants.

[77] Cui G., Sun F., Gao X., Xie K., Zhang C., Liu S., Xi Y. (2018). Proteomic analysis of melatonin-mediated osmotic tolerance by improving energy metabolism and autophagy in wheat (*Triticum aestivum* L.). Planta.

[78] Chen J., Zhang Y., Yin H., Liu W., Hu X., Li D., Lan C., Gao L., He Z., Cui F. (2022). The pathway of melatonin biosynthesis in common wheat (*Triticum aestivum* L.). J. Pineal Res..

[79] Yang X., Chen J., Ma Y., Huang M., Qiu T., Bian H., Han N., Wang J. (2022). Function, Mechanism, and Application of Plant Melatonin: An Update with a Focus on the Cereal Crop, Barley (*Hordeum vulgare* L.). Antioxidants.

[80] Arnao M.B., Hernandez-Ruiz J. (2009). Chemical stress by different agents affects the melatonin content of barley roots. J. Pineal Res..

[81] Arnao M.B., Hernandez-Ruiz J. (2009). Protective effect of melatonin against chlorophyll degradation during the senescence of barley leaves. J. Pineal Res..

[82] Chang T.-l., Xi Q.-q., Wei X.-y., Xu L., Wang Q.-q., Fu J.-y., Ling C., Zuo Y., Zhao Y., He H.-y. (2022). Rhythmical redox homeostasis can be restored by exogenous melatonin in hulless barley (*Hordeum vulgare* L.var. nudum) under cold stress. Environ. Exp. Bot..

[83] Chang T., Zhao Y., He H., Xi Q., Fu J., Zhao Y. (2021). Exogenous melatonin improves growth in hulless barley seedlings under cold stress by influencing the expression rhythms of circadian clock genes. Peerj.

[84] Tian X., He X., Xu J., Yang Z., Fang W., Yin Y. (2022). Mechanism of calcium in melatonin enhancement of functional substance-phenolic acid in germinated hulless barley. Rsc Adv..

[85] Danilova E.D., Zlobin I.E., Kuznetsov V.V., Efimova M.V. (2021). Exogenic Melatonin Reduces the Toxic Effect of Polymetallic Stress on Barley Plants. Dokl. Biochem. Biophys..

[86] Barman D., Ghimire O.P., Chinnusamy V., Kumar R.R., Arora A. (2019). Amelioration of heat stress during reproductive stage in rice by melatonin. Indian J. Agric. Sci..

[87] Fan X., Zhao J., Sun X., Zhu Y., Li Q., Zhang L., Zhao D., Huang L., Zhang C., Liu Q. (2022). Exogenous melatonin improves the quality performance of rice under high temperature during grain filling. Agronomy.

[88] Barman D., Kumar R., Ghimire O.P., Ramesh R., Gupta S., Nagar S., Pal M., Dalal M., Chinnusamy V., Arora A. (2024). Melatonin induces acclimation to heat stress and pollen viability by enhancing antioxidative defense in rice (*Oryza sativa* L.). Environ. Exp. Bot..

[89] Chen X., Laborda P., Liu F. (2020). Exogenous Melatonin Enhances Rice Plant Resistance Against Xanthomonas oryzae pv. oryzae. Plant Dis..

[90] Huang Y., Jiang H., Wang N., Liu Y., Hu H. (2018). Effects of exogenous melatonin on the growth of rice seedlings under As stress. Chin. J. Ecol..

[91] Jan R., Asif S., Asaf S., Du X.-X., Park J.-R., Nari K., Bhatta D., Lee I.-j., Kim K.-M. (2023). Melatonin alleviates arsenic (As) toxicity in rice plants via modulating antioxidant defense system and secondary metabolites and reducing oxidative stress. Environ. Pollut..

[92] Yan F., Wei H., Ding Y., Li W., Liu Z., Chen L., Tang S., Ding C., Jiang Y., Li G. (2021). Melatonin regulates antioxidant strategy in response to continuous salt stress in rice seedlings. Plant Physiol. Biochem..

[93] Sher A., Hassan M.U., Sattar A., Ul-Allah S., Ijaz M., Hayyat Z., Bibi Y., Hussain M., Qayyum A. (2023). Exogenous application of melatonin alleviates the drought stress by regulating the antioxidant systems and sugar contents in sorghum seedlings. Biochem. Syst. Ecol..

[94] Hwang O.J., Back K. (2022). Exogenous Gibberellin Treatment Enhances Melatonin Synthesis for Melatonin-Enriched Rice Production. Biomolecules.

[95] Byeon Y., Lee H.Y., Lee K., Park S., Back K. (2014). Cellular localization and kinetics of the rice melatonin biosynthetic enzymes SNAT and ASMT. J. Pineal Res..

[96] Liu J., Shabala S., Zhang J., Ma G., Chen D., Shabala L., Zeng F., Chen Z.H., Zhou M., Venkataraman G. (2020). Melatonin improves rice salinity stress tolerance by NADPH oxidase-dependent control of the plasma membrane K+ transporters and K+ homeostasis. Plant Cell Environ..

[97] Byeon Y., Park S., Lee H.Y., Kim Y.-S., Back K. (2014). Elevated production of melatonin in transgenic rice seeds expressing rice tryptophan decarboxylase. J. Pineal Res..

[98] Byeon Y., Back K. (2014). Melatonin synthesis in rice seedlings in vivo is enhanced at high temperatures and under dark conditions due to increased serotonin N-acetyltransferase and N-acetylserotonin methyltransferase activities. J. Pineal Res..

[99] Hwang O.J., Kang K., Back K. (2020). Effects of Light Quality and Phytochrome Form on Melatonin Biosynthesis in Rice. Biomolecules.

[100] Arnao M.B., Hernández-Ruiz J. (2014). Melatonin: Plant growth regulator and/or biostimulator during stress?. Trends Plant Sci..

[101] Tripathi D.K., Singh V.P., Brestic M., Deshmukh R., Vaculik M. (2022). Priming-mediated abiotic stress management in plants: Recent avenues and future directions. Plant Stress.

[102] Zou C., Li L., Miki D., Li D., Tang Q., Xiao L., Rajput S., Deng P., Peng L., Jia W. (2019). The genome of broomcorn millet. Nat. Commun..

[103] Rajput S.G., Santra D.K., Schnable J. (2016). Mapping QTLs for morpho-agronomic traits in proso millet (*Panicum miliaceum* L.). Mol. Breed..

[104] Zhang R., Yue Z., Chen X., Wang Y., Zhou Y., Xu W., Huang R. (2022). Foliar applications of urea and melatonin to alleviate waterlogging stress on photosynthesis and antioxidant metabolism in sorghum seedlings. Plant Growth Regul..

[105] Liang J., Wusiman M., Fang Z. (2021). Effects of Exogenous Growth Substances on Seed Germination of Sweet Sorghum under Drought Stress. Acta Agrestia Sin..

[106] Koo A.J., Arimura G.-i. (2022). Molecular biology of chemical defenses. Plant Mol. Biol..

[107] Ren J., Yang X., Ma C., Wang Y., Zhao J. (2021). Melatonin enhances drought stress tolerance in maize through coordinated regulation of carbon and nitrogen assimilation. Plant Physiol. Biochem..

[108] Wang Y., Wang J., Guo H., Wu X., Hao M., Zhang R. (2023). Integrative transcriptome and metabolome analysis reveals the mechanism of exogenous melatonin alleviating drought stress in maize roots. Plant Physiol. Biochem..

[109] Wang J., Lv P., Yan D., Zhang Z., Xu X., Wang T., Wang Y., Peng Z., Yu C., Gao Y. (2022). Exogenous Melatonin Improves Seed Germination of Wheat (*Triticum aestivum* L.) under Salt Stress. Int. J. Mol. Sci..

[110] Niu Y., Li J., Sun F., Song T., Han B., Liu Z., Su P. (2023). Comparative transcriptome analysis reveals the key genes and pathways involved in drought stress response of two wheat (*Triticum aestivum* L.) varieties. Genomics.

[111] Huangfu L., Zhang Z., Zhou Y., Zhang E., Chen R., Fang H., Li P., Xu Y., Yao Y., Zhu M. (2021). Integrated physiological, metabolomic and transcriptomic analyses provide insights into the roles of exogenous melatonin in promoting rice seed germination under salt stress. Plant Growth Regul..

[112] Zhao C., Yang M., Wu X., Wang Y., Zhang R. (2021). Physiological and transcriptomic analyses of the effects of exogenous melatonin on drought tolerance in maize (*Zea mays* L.). Plant Physiol. Biochem..

[113] Zhao H., Su T., Huo L., Wei H., Jiang Y., Xu L., Ma F. (2015). Unveiling the mechanism of melatonin impacts on maize seedling growth: Sugar metabolism as a case. J. Pineal Res..

[114] Ren J., Yang X., Zhang N., Feng L., Ma C., Wang Y., Yang Z., Zhao J. (2022). Melatonin alleviates aluminum-induced growth inhibition by modulating carbon and nitrogen metabolism, and reestablishing redox homeostasis in *Zea mays* L.. J. Hazard. Mater..

[115] Yang X., Ren J., Lin X., Yang Z., Deng X., Ke Q. (2023). Melatonin Alleviates Chromium Toxicity in Maize by Modulation of Cell Wall Polysaccharides Biosynthesis, Glutathione Metabolism, and Antioxidant Capacity. Int. J. Mol. Sci..

[116] Wang J., Gao X., Wang X., Song W., Wang Q., Wang X., Li S., Fu B. (2022). Exogenous melatonin ameliorates drought stress in Agropyron mongolicum by regulating flavonoid biosynthesis and carbohydrate metabolism. Front. Plant Sci..

[117] Wang W., Guo J., Ma J., Wang Z., Zhang L., Wang Z., Meng M., Zhang C., Sun F., Xi Y. (2023). Comprehensive Transcriptomic and Metabolic Profiling of Agrobacterium-tumefaciens-Infected Immature Wheat Embryos. Int. J. Mol. Sci..

[118] Lee C., Chung C.-T., Hong W.-J., Lee Y.-S., Lee J.-H., Koh H.-J., Jung K.-H. (2021). Transcriptional changes in the developing rice seeds under salt stress suggest targets for manipulating seed quality. Front. Plant Sci..

[119] Xie Z., Wang J., Wang W., Wang Y., Xu J., Li Z., Zhao X., Fu B. (2021). Integrated analysis of the transcriptome and metabolome revealed the molecular mechanisms underlying the enhanced salt tolerance of rice due to the application of exogenous melatonin. Front. Plant Sci..

[120] Liang C., Zheng G., Li W., Wang Y., Hu B., Wang H., Wu H., Qian Y., Zhu X.-G., Tan D.-X. (2015). Melatonin delays leaf senescence and enhances salt stress tolerance in rice. J. Pineal Res..

[121] Yin Y., Xu J., He X., Yang Z., Fang W., Tao J. (2022). Role of exogenous melatonin involved in phenolic acid metabolism of germinated hulless barley under NaCl stress. Plant Physiol. Biochem..

[122] Zhang G., Yan Y., Zeng X., Wang Y., Zhang Y. (2022). Quantitative Proteomics Analysis Reveals Proteins Associated with High Melatonin Content in Barley Seeds under NaCl-Induced Salt Stress. J. Agric. Food Chem..

[123] Awan S.A., Khan I., Rizwan M., Irshad M.A., Xiaosan W., Zhang X., Huang L. (2023). Reduction in the cadmium (Cd) accumulation and toxicity in pearl millet (*Pennisetum glaucum* L.) by regulating physio-biochemical and antioxidant defense system via soil and foliar application of melatonin. Environ. Pollut..

[124] Wang Y.F., Guo Y.Y., Zhao C.F., Li H.J., Zhang R.H. (2021). Exogenous Melatonin Achieves Drought Tolerance by Improving Photosynthesis in Maize Seedlings Leaves. Russ. J. Plant Physiol..

[125] Arnao M.B., Hernández-Ruiz J. (2018). Melatonin and its relationship to plant hormones. Ann. Bot..

[126] Zhang N., Sun Q., Zhang H., Cao Y., Weeda S., Ren S., Guo Y.-D. (2015). Roles of melatonin in abiotic stress resistance in plants. J. Exp. Bot..

[127] Desouhant E., Gomes E., Mondy N., Amat I. (2019). Mechanistic, ecological, and evolutionary consequences of artificial light at night for insects: Review and prospective. Entomol. Exp. Appl..

