# Supplementary material for: Melatonin-Mediated Molecular Responses in Plants: Enhancing Stress Tolerance and Mitigating Environmental Challenges in Cereal Crop Production
Source: Int J Mol Sci. 2024 Apr 21;25(8):4551. doi: 10.3390/ijms25084551 (PMC11049982; doi:10.3390/ijms25084551)
Supplement: Supplementary file 1 [file ijms-25-04551-s001.zip › ijms-2931285-supplementary.pdf]

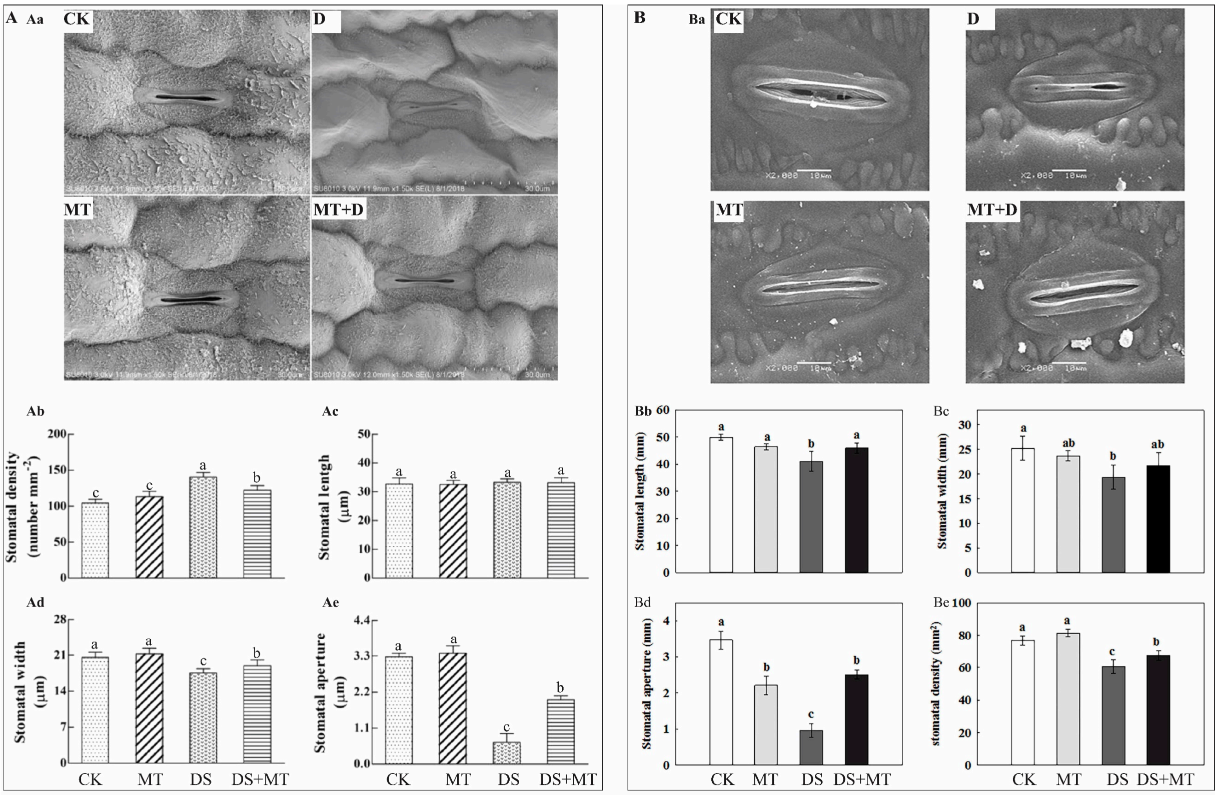

Figure S1. The effects of melatonin on stomatal traits in polyethylene glycol (PEG) treated maize seedling leaves were evaluated (A). Images of seedling leaves were analyzed using scanning electron microscopy (SEM) on the third day of drought treatment (Aa). Under various conditions, the density (Ab), length (Ac), width (Ad), and aperture (Ae) of stomata were measured. CK, Stomata from Hoagland solution only; D, Stomata from drought-treated plants grown in Hoagland solution with 20% PEG-6000; MT, Stomata from plants with Hoagland solution (100 μmol melatonin L^{-1}); MT+DT, Stomata from melatonin plus drought treatment (100 μmol melatonin L^{-1} with 20% PEG-6000 in Hoagland solution) [63]. Effect of exogenous melatonin and drought stress on stomatal traits (B). Stomata from well-watered (CK). Stomata from drought (D). Stomata from well-watered + 100 μM melatonin (MT). Stomata from drought + 100 μM melatonin (MT+DT). Stomatal length (Bb), width (Bc), aperture (Bd), and density (Be). The data shown are the mean ± SD of three replications. Different lowercase letters indicate significant differences ($P < 0.05$) according to Duncan's multiple range tests [72].

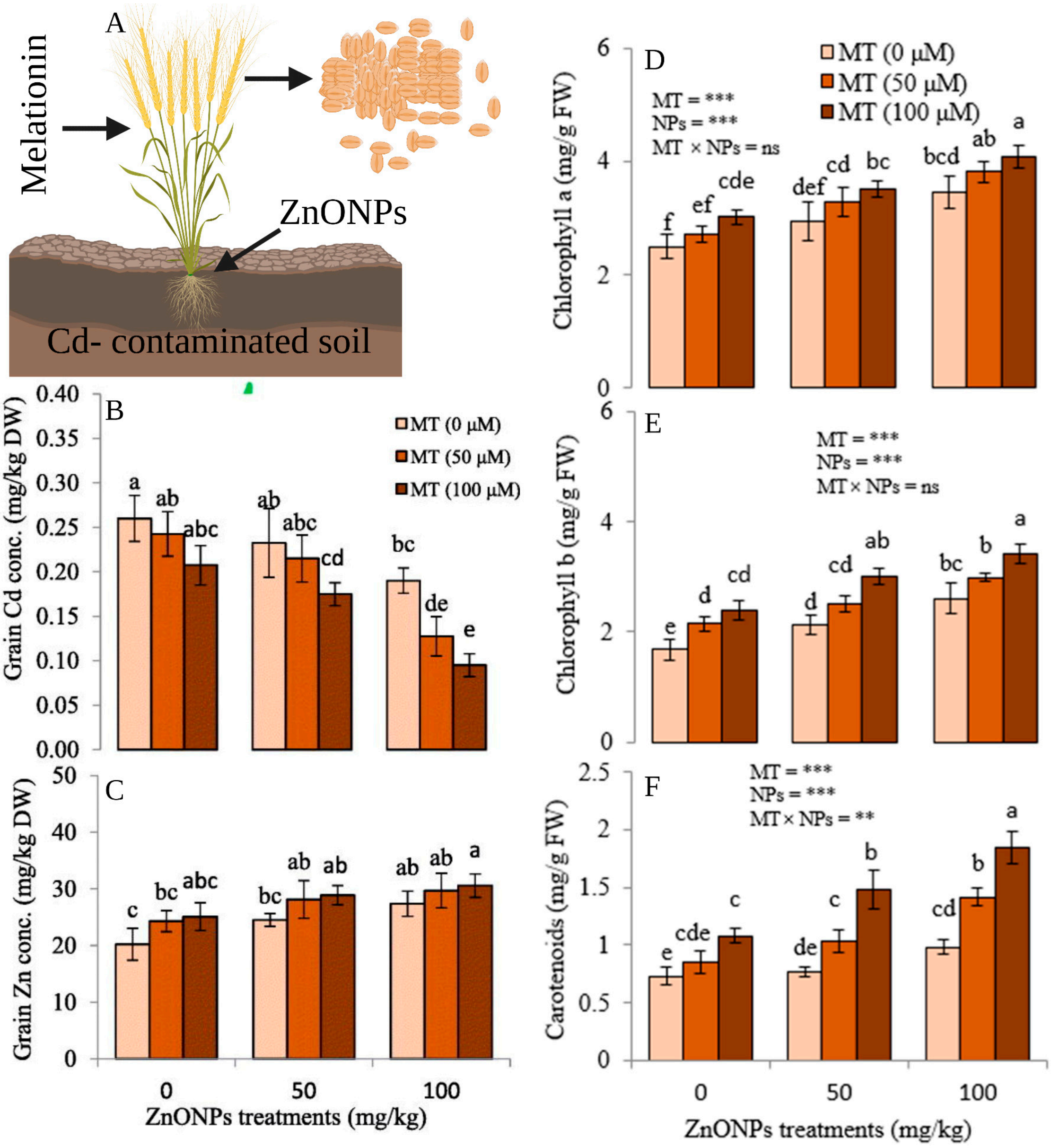

Figure S2. Effect of ZnONPs alone and combined with melatonin (A) on Cd concentrations in grains (B), Zn concentration in grain (C), chlorophyll a (D), chlorophyll b (E) and carotenoid concentrations (F) in leaves of wheat plants under cadmium stress (Cd). The data presented in the figure are mean values of four replications of each treatment along with standard deviation (SD). Different lowercase letters given on the bars demonstrate the significant difference among the treatments at $p \leq 0.05$. Asterisks indicate statistical significance where $^{**}p \leqslant 0.01$, $^{***}p \leqslant 0.001$, and ns indicates not significant. Reproduced with permission of [40] © (2023) Elsevier.

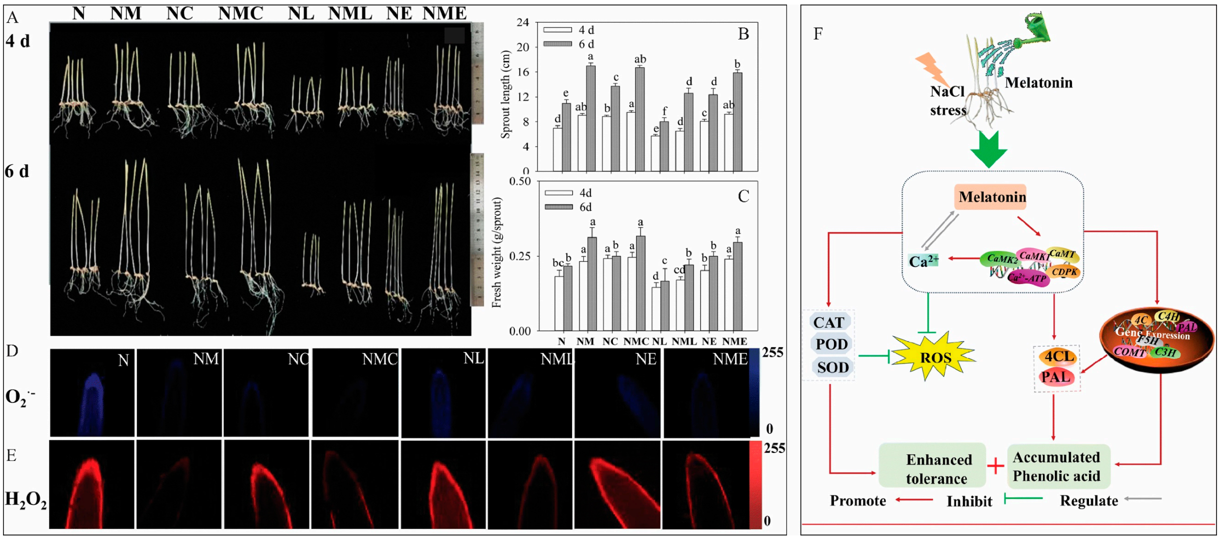

Figure S3. Effects of melatonin and Ca^{2+} on the (A) growth performance, (B) sprout length, (C) fresh weight, and root tip staining of (D) O_2^-, (E) H_2O_2, and (F) phenolic acid biosynthesis and enhanced tolerance in barley sprout under salinity stress. The scale length is 100 mm. Different lowercase letters indicate significant differences among the treatments at the same germination time using Tukey's test ($P < 0.05$). N: NaCl; NM: NaCl + MT; NC: NaCl + $CaCl_2$; NMC: NaCl +MT + $CaCl_2$; NL: NaCl + $LaCl_3$; NML: NaCl + MT + $LaCl_3$; NE: NaCl + EGTA; NME: NaCl + MT + EGTA [84].

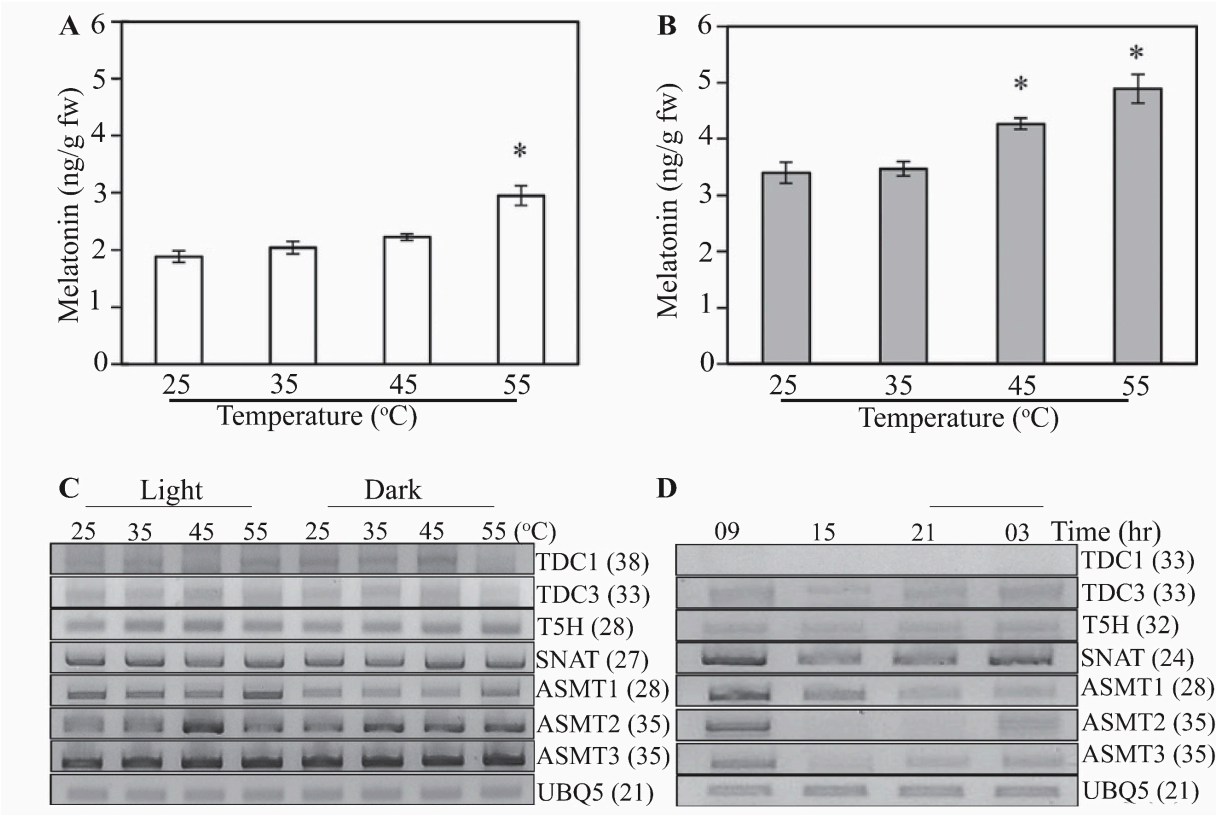

Figure S4. Melatonin levels (A and B) and gene expression (C and D) were analyzed under varying high temperatures in rice seedlings. Measurements were taken under both light (A) and dark conditions (B) using HPLC for melatonin levels and RT-PCR for melatonin biosynthetic genes (C and D). Significant differences were observed compared to the 25°C treatment. ASMT, N-acetylserotonin methyltransferase; TDC, tryptophan decarboxylase; T5H, tryptamine 5-hydroxylase; SNAT, serotonin N-acetyltransferase; UBQ5, rice ubiquitin-5. The numbers in parentheses represent the total number of PCR cycles. The data represent the values of three replicated ±S.D. Asterisks (*) indicate statistically significant deviations from the 25°C treatment ($P < 0.05$). Reproduced with permission of [98] © (2014) Wiley.
